# Case report: The effective response to pembrolizumab in combination with bevacizumab in the treatment of a recurrent glioblastoma with multiple extracranial metastases

**DOI:** 10.3389/fonc.2022.948933

**Published:** 2022-08-16

**Authors:** Gang Yang, Yu Fang, Ming Zhou, Wei Li, Dapeng Dong, Jing Chen, Yong Da, Kunpeng Wang, Xinru Li, Xiaoyan Zhang, Tonghui Ma, Ge Shen

**Affiliations:** ^1^ Oncology Department, Beijing Fengtai You’anmen Hospital, Beijing, China; ^2^ Genetron Health (Beijing) Technology, Co. Ltd., Beijing, China; ^3^ Oncology Department, Beijing Hui’an TCM-Integrated Hospital, Beijing, China

**Keywords:** extracranial metastases glioblastoma, bevacizumab, pembrolizumab, combined treatment, case report

## Abstract

Multiple extracranial metastases of recurrent glioblastoma are rare and often indicate a very poor prognosis. The main conventional treatments are chemotherapy, radiotherapy, chemoradiotherapy or antiangiogenic therapy. Median overall survival is 2.3 to 6 months after the detection of extracranial metastases, and to date, there is no effective treatment for these patients. Herein, we report a recurrent glioblastoma patient with lung metastasis treated with a combination therapy containing bevacizumab and pembrolizumab due to overexpression of PD-L1 and the absence of driver mutations. The progression-free survival was 11 months from lung metastases to bone metastases. This combination treatment was further used as maintenance therapy for another 11 months after bone metastasis and secondary dorsal metastasis because there was no suitable treatment alternative. The overall survival was 27 months after lung metastases, which is much longer than previously reported cases. To our knowledge, this was the first effective use of bevacizumab plus pembrolizumab in a glioblastoma patient with extracranial metastases. Furthermore, this was the first time that bevacizumab plus pembrolizumab was used as a maintenance treatment in glioblastoma, with 11 months of response. Importantly, we showed that such combination therapy may be a novel and effective therapy for glioblastoma patients with extracranial metastases.

## Introduction

Glioblastoma (GBM) is the most common primary malignant brain tumor, but despite advances in treatment strategies, the prognosis remains poor (1, 2). The mainstay first-line treatment of GBM is surgery followed by postoperative adjuvant treatment using chemoradiotherapy. The U.S. Food and Drug administration (FDA) has also approved temozolomide (TMZ) to treat primary GBM, but it has a relatively poor response rate (3). Approximately 90% of GBM patients experience postoperative recurrence (4). Bevacizumab, an angiogenesis inhibitor, is approved for recurrent GBM multiforme, but the median duration of response is approximately 3.9 months, and the 6-month progression-free survival (PFS) rate is approximately 36.0% (5). In recent years, immune checkpoint inhibitors (e.g., nivolumab and pembrolizumab) and/or targeted inhibitors (such as BRAF inhibitors, mTOR inhibitors, and MET inhibitors) have improved the outcomes of patients with GBM (6, 7). Interestingly, the combination of antiangiogenic drugs and immunotherapy yielded good results when tested in driver mutation-absent cancers (8, 9). This synergistic effect could be explained by the antiangiogenic agents that can normalize the abnormal blood vessels, which may convert the tumor microenvironment and thus improve the effects of immune checkpoint inhibitors (10). However, a phase II clinical trial showed limited benefits from pembrolizumab combined with bevacizumab for recurrent GBM with a reported median overall survival (mOS) of 10.3 months (11). It is unclear whether GBM cells developed resistance to pembrolizumab and bevacizumab, or whether the intracranial environment limited their effects.

Extracranial metastasis from GBM is very rare, with an incidence of approximately 0.4–0.5% (12–14). There is still no consensus on the standard treatment for GBM extracranial metastases; therefore, most patients receive radiotherapy, chemotherapy, or bevacizumab (15–18). However, a meta-analysis showed that the mOS of GBM patients from extracranial metastases diagnosis to death was only 2.3 months (19).

In this case, we report a recurrent GBM case with lung metastases. Considering the poor response of lung lesions to the combination treatment of bevacizumab and TMZ and positive PD-L1 expression of the lesion, the immune inhibitor pembrolizumab was added to bevacizumab for the patient’s subsequent treatment, with a PFS of 11 months. Then the patient received the same maintenance treatment for 11 months. The OS was approximately 27 months from the day of extracranial lung metastasis detection to death, which was much longer than previous treatment modalities.

## Case report

**Figure 1:**
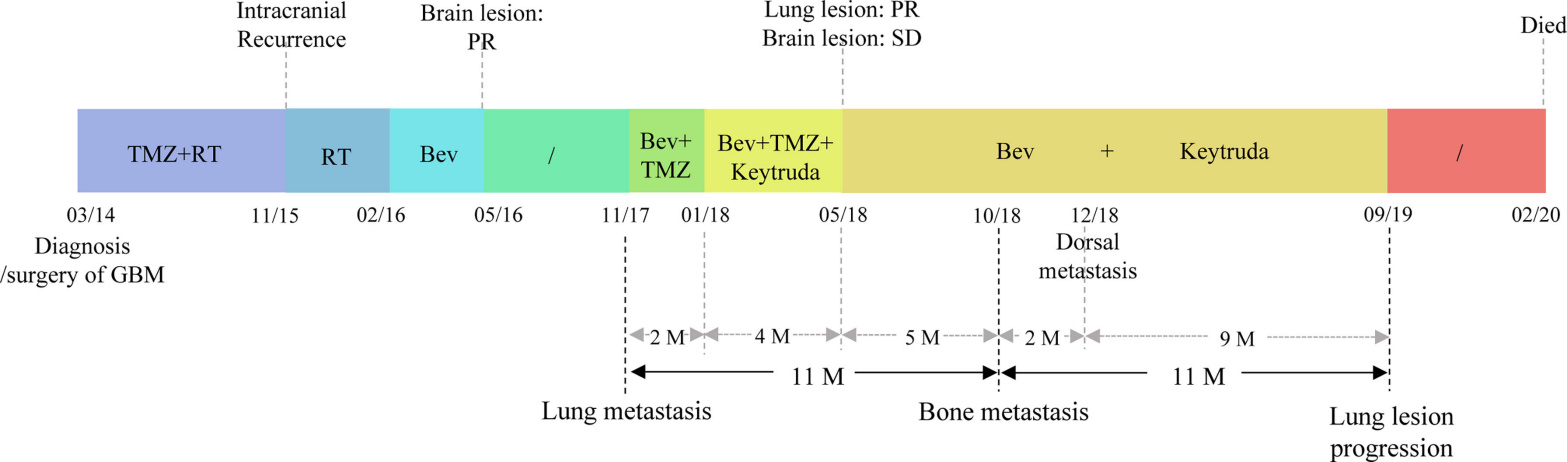
The treatment timeline of the patient. The patient was diagnosed with glioblastoma in March 2014. He underwent surgery, followed by standard adjuvant treatment with Temozolomide (TMZ) and radiotherapy (RT). The tumor recurred intracranially in November 2015, and he received RT and Bevacizumab (Bev) treatment, the response evaluation of the brain lesion was a partial response (PR). In November 2017, lung metastases were detected, the patient accepted Bev and TMZ, but the lung lesion progressed. Two months (M) later, Keytruda was added because of a positive PD-L1 expression and a partial response (PR) was achieved for the metastatic lesion while the brain lesion was a stable disease (SD). Then TMZ was discontinued to relieve the symptoms of fatigue and due to the unmethylation of the MGMT promoter (May 2018). The patients received five months of Bev plus Keytruda. In October 2018, bone metastases were identified. The patient continued treatment with Bev and Keytruda for two months because no other treatment could be used. Dorsal metastasis was found in December 2018 and due to no other suitable treatment, and the patient continued to receive Bev and Keytruda for another nine months before pulmonary lesion progression. Five months later, the patient discontinued all treatment and died from a pulmonary infection.

A 58-year-old man without family disease history was admitted to our hospital in March 2014, complaining of left fingertip numbness for more than two months, intermittent dizziness and left limb weakness for half a month (**Figure 1**). Magnetic resonance imaging (MRI) and positron emission tomography/computed tomography (PET/CT) of the brain revealed multiple lesions in the right lobe (**Figure S1A**). As a result, the patient underwent brain surgery, and the pathological analysis of the tumor revealed GBM with the presence of oligodendroglioma components. Immunohistochemistry (IHC) results demonstrated positive staining for vascular endothelial growth factor (VEGF) and glial fibrillary acidic protein (GFAP), partial positive expression for Ki67 and oligodendrocyte lineage transcription factor 2 (OLIG2). The 1p19q codeletion was not detected by fluorescence *in situ* hybridization. The *IDH1/2* gene was wild-type, and the O^6^-methylguanine-DNA methyltransferase (MGMT) promoter was unmethylated. The patient received standard postoperative radiotherapy (Dt6000cGy/30f/6w) with TMZ (160 mg d1-5). A follow-up MRI in April 2015 showed the absence of new lesions (**Figure S1B**). However, a follow-up in November 2015 revealed right temporal and right occipital recurrence (**Figures S1C, D**). Subsequently, the patient underwent a radiotherapy regimen (Dt: 30 Gy/5 f) followed by four cycles of bevacizumab (5 mg/kg, d1, q3w) and then achieved a RECIST criteria partial response (PR). At the same time, the patient showed an improvement in quality of life.

**Figure 2:**
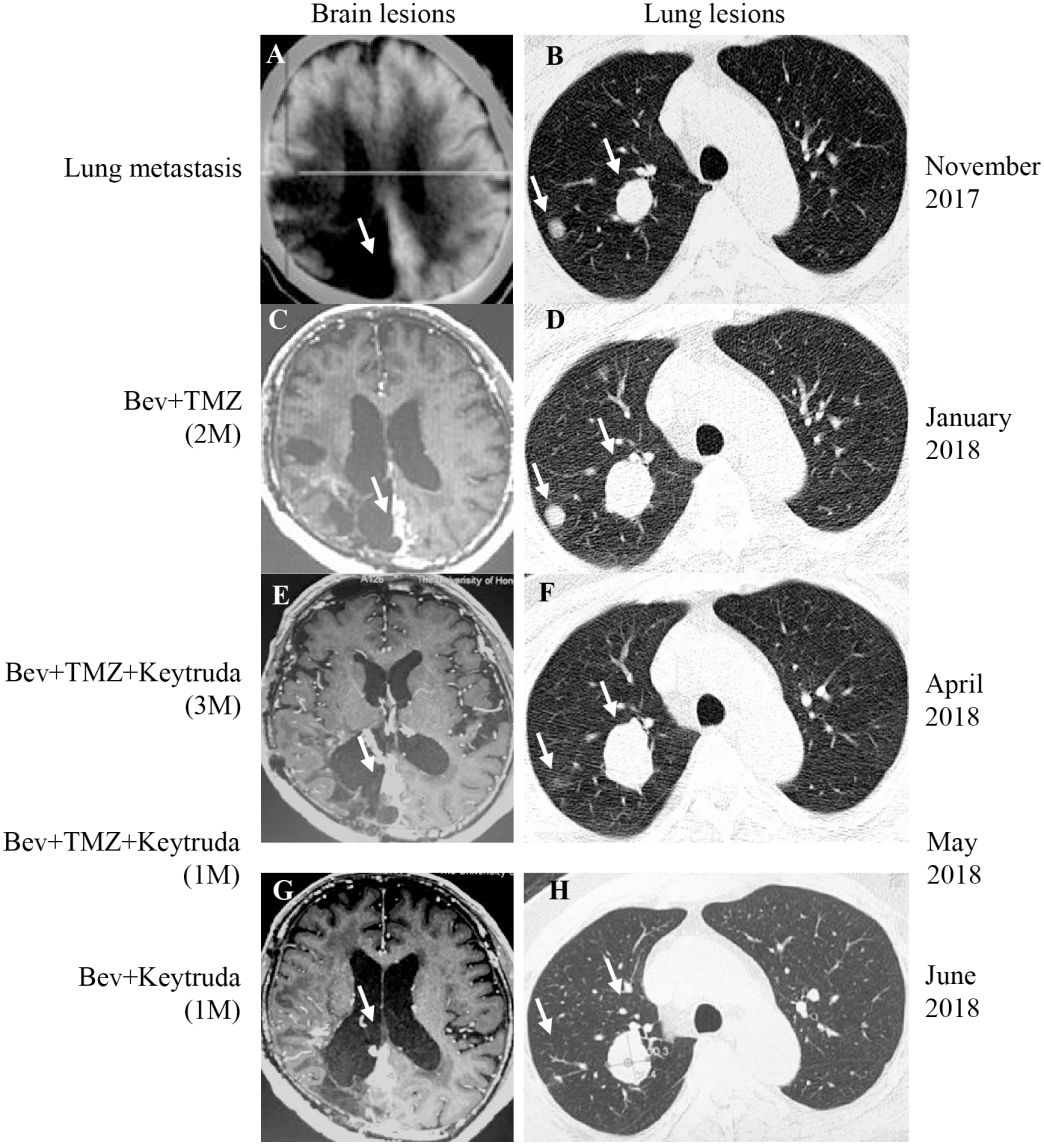
PET-CT/MRI images of the brain and lung at the time of lung metastasis, during and after treatment. **(A, B)** The brain lesion was stable and the lung metastasis was found in November 2017. **(C, D)** After two months of Bevacizumab (Bev)+TMZ treatment, the brain lesion was stable and the lung lesions progressed (January 2018). **(E, F)** After Keytruda was added for three months (April 2018), the brain lesion was stable, and the response evaluation of lung lesion was PR. **(G, H)** After Keytruda was added for five months (June 2018), the brain lesion was kept on SD and the lung lesion was kept on PR. The white arrow represents the lesion. M, month.

**Figure 3:**
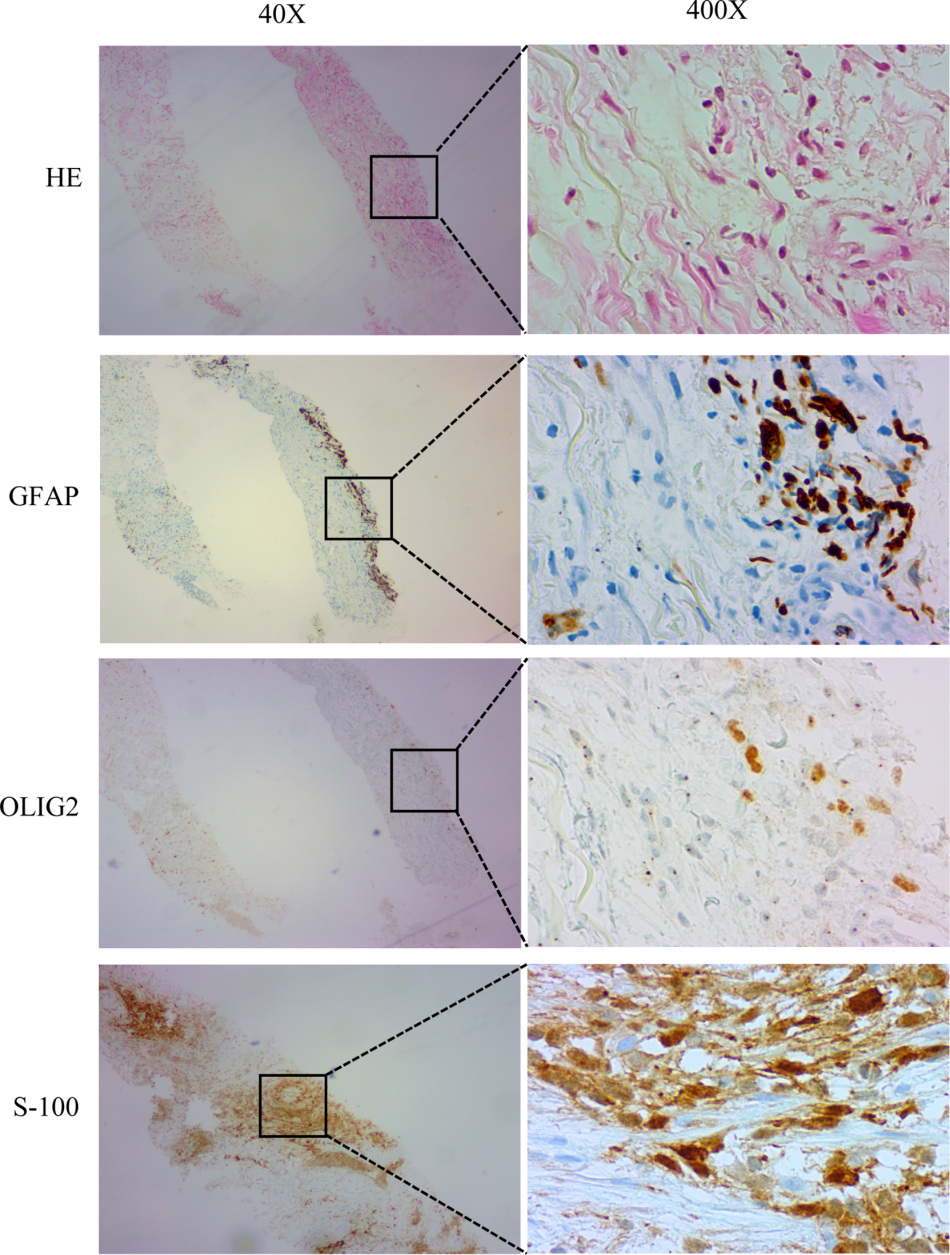
Histopathology and immunostaining of metastatic lung lesions. Hematoxylin-Eosin (HE) staining showed that the lung lesion was neoplastic. Immunohistochemistry (IHC) showed positive GFAP, OLIG2 and S-100. Magnification: 40X and 400X.

In November 2017, the patient returned to our hospital complaining of chronic cough. PET/CT showed that the brain lesion (**Figure 2A**) was stable when compared with the brain lesion in November 2015 (**Figure S1D**), but multiple nodules were observed in the right lung (**Figure 2B**, November 2017). Lung biopsy was performed, and we confirmed that the lung lesion was, in fact, a neoplastic metastasis of GBM by pathological analysis and IHC (**Figure 3**). The results of IHC was positive staining of GFAP, OLIG2, and S-100 protein was also observed. Due to no standard treatment for extracranial metastases GBM, the next-generation sequencing (NGS) and PD-L1 detection were performed on lung lesions, expecting to find a treatment target. The NGS results from Onco PanScan™ (GenetronHealth) revealed *KRAS* mutation (**Figure S2A**), *TP53* mutation (**Figures S2B, C**) and *FGFR3* fusion (**Figure S2D**) but the absence of *IDH1/2* mutation, *H3F3A/HIST1H3B* mutation and *RELA* mutation (**Table 1**). As there was no suitable targeted therapy at the time, bevacizumab and TMZ was used for recurrent GBM according to the NCCN guidelines; thus, the patient underwent two months of bevacizumab (300 mg, q3w, 4 cycles; 300 mg, q4w, 2 cycles) and TMZ (420 mg, q28d) treatment. The brain lesions remained stable, while the lung lesions increased slightly in size (**Figures 2C, D**, January 2018 vs. November 2017). Fortunately, the lung lesion had positive expression of PD-L1; therefore, we decided to add pembrolizumab with bevacizumab and TMZ to our treatment strategy. The treatment plan of pembrolizumab was as follows: 200 mg q2w, 2 cycles; then 200 mg, q3w, 1 cycle; then 200 mg, q4w 2 cycles. During this treatment, we reassessed the patient in April 2018, and the MRI scan revealed an effective response of the lung lesion, with a RECIST criteria PR, while the brain lesion was stable (**Figures 2E, F**, April 2018 vs. January 2018). Because the patient was suffering from cancer-related fatigue and the MGMT promoter was unmethylated, we discontinued TMZ in May 2018. From then on, the patient was on bevacizumab plus pembrolizumab only. Interestingly, the lung lesion decreased further in size while the brain lesion remained stable (**Figures 2G, H**, June 2018 vs. April 2018). However, bone metastases appeared four months later (Octoble 2018). There was a total of 11 months (November 2017 to October 2018) of remission from lung to bone metastases, during which bevacizumab and pembrolizumab were used (**Figure 1**).

**Table 1:** NGS gene mutation profiling in lung specimen.

Gene	Mutation type	c.dot	Genovariation
*KRAS*	Missense mutation	c.40G>A	p.V14I
*TP53*	Nonsense mutation	c.637C>T	p.R213*
*TP53*	Missense mutation	c.818G>A	p.R273H
*FGFR3*	Gene fusion	/	FGFR3-TACC3
*IDH1/IDH2*	/	/	/
*H3F3A/HIST1H3B*	/	/	/
*RELA*	/	/	/

NGS, next-generation sequencing.

Following bone metastases, the patient agreed to continue using bevacizumab (300 mg, q4w) and pembrolizumab (200 mg, q4w) as maintenance therapy due to the unavailability of suitable alternative treatment. Nearly two months later (December 2018), a left posterior dorsal mass was found during routine follow-up (**Figure 1**). Nonetheless, the patient was kept on the same regimen for nine months with no suitable treatment available. After September 2019, due to the lung lesion progression, the patient was in poor physical condition, could not tolerate further treatment, and died from a lung infection in February 2020. The total survival time of the patient from extracranial metastases of the lung to death was approximately 27 months.

## Discussion

Extracranial metastasis from GBM is extremely rare. Currently, there is no standard guideline for the treatment of extracranial metastasis from GBM. In some previous reports, GBM patients with extracranial metastases generally received chemotherapy, radiotherapy, radiochemotherapy, or chemotherapy combined with bevacizumab and had a very short survival time after metastasis (15–18, 20). The patient in our case received multiple lines of treatment after lung metastasis was detected. Because the disease progressed after bevacizumab and TMZ as first line treatment, immunotherapy plus bevacizumab treatment was administered as subsequent treatment, which resulted a long survival of 27 months. The OS of the patient from diagnosis to death was approximately 70 months, which is much longer than previous reports. There are three important findings from our case. First, a combination treatment of pembrolizumab and bevacizumab after lung metastasis was shown to be effective since the lung lesion achieved PR and the brain lesion was SD. This suggests that the treatment of immunotherapy plus angiogenesis inhibition is potent against GBM cells that metastasize to the lungs. Previously, a phase II clinical trial showed a limited benefit of 10.3 months of OS from pembrolizumab combined with bevacizumab for recurrent GBM (11). It was unclear whether the poor response was due to the glioma cells being insensitive to the drugs, whether the blood-brain barrier limited drug entry, or whether the tumor environment affected the response. Here, our results reveal that there is a strong case for the latter two reasons. Second, we found that GBM patient in China and other countries was similar in gender ratio (63.0% male vs. 65.0% male), treatment modalities (radiotherapy and chemotherapy), MGMT promoter methylation ratio (44.7% vs. 54.6%) and OS (14 months vs.15.7 months) (21, 22). Furthermore, we compared the characteristics of patients with GBM extracranial metastases in China and other countries, and found that metastasis site, treatment modalities and the survival time were similar (19, 23, 24). A cases series of extracranial metastatic GBM reported by MSKCC showed that the median survival time after metastasis was 5 months (23), and a case report about a Chinese extracranial metastatic GBM showed that the survival time was 6 months from extracranial metastases (24). In our case, after receiving a combination of bevacizumab and pembrolizumab, the total survival reached 27 months from extracranial lung metastasis diagnosis to death, which was longer than previous reports. Third, our patient used pembrolizumab in combination with bevacizumab as a maintenance treatment for two months after a secondary metastasis to the bone was detected. This treatment was maintained for another nine months, even after dorsal metastases were localized. A retrospective study showed that patients treated with immune checkpoint inhibitors could benefit from adopting the same treatment regimen after progression, with a median PFS of 2.7 months and a mOS of 7 months from the first progression to the second progression (25). Herein, the patient progressed slowly, the combination therapy of angiogenesis and immune checkpoint inhibitors was continued regardless of progression, and the patient achieved prolonged OS (27 months after lung metastasis). Similarly, our study showed that using the previously effective treatment as a maintenance treatment is a choice for patients with no alternative treatment options after progression.

In our case, NGS and IHC detection were performed on the patient’s lung biopsy tissue at the time of lung metastasis. The results showed KRAS and TP53 mutations, FGFR3-TACC3 fusion and PD-L1 positivity. Only PD-L1 inhibitors were approved at that time; therefore, the patient received the PD-L1 inhibitor, pembrolizumab, as an add-on therapy. Additionally, as mentioned above, the *FGFR3-TACC3* fusion was detected from the lung biopsy tissue. A clinical trial confirmed an effective response to erdafitinib in GBM patients with the FGFR3-TACC3 fusion (26). Unfortunately, the targeted drug erdafitinib for FGFR2 or FGFR3 genetic alterations was not approved by the National Medical Products Administration at the time and the FDA only approved it for metastatic urothelial carcinoma in April 2019. More clinical trials are warranted to broaden the indications of erdafitinib to encompass GBM patients in the future. Nevertheless, some targeted drugs, including those targeting fusion mutations, such as NTRK inhibitors, have been approved for solid tumors, hence the importance of performing multigene detection in GBM or recurrent GBM to provide more opportunities for these patients.

Herein, our GBM patient underwent various detection and treatment modalities, and he was ultimately treated with bevacizumab combined with pembrolizumab after extracranial lung metastasis was detected. The combination treatment haltered the progression of the disease and improved the patient’s OS time considerably. In light of our findings, we provide a valuable reference for treating other GBM patients with extracranial metastasis in the future.

## Data availability statement

The original contributions presented in the study are included in the article/**Supplementary Material**. Further inquiries can be directed to the corresponding authors.

## Ethics statement

Written informed consent was obtained from the individual(s) for the publication of any potentially identifiable images or data included in this article.

## Author contributions

GS, TM, and XZ contributed to conception and design of the study. GY, WL, XL, KW and JC prepared figures and background research. YF wrote the first draft of the manuscript. YD, MZ, and DD wrote sections of the manuscript. All authors contributed to the article and approved the submitted version.

## Conflict of interest

Author YF, WL, JC, XZ and TM are employed by Genetron Health (Beijing) Technology, Co. Ltd.

The remaining authors declare that the research was conducted in the absence of any commercial or financial relationships that could be construed as a potential conflict of interest.

## Publisher’s note

All claims expressed in this article are solely those of the authors and do not necessarily represent those of their affiliated organizations, or those of the publisher, the editors and the reviewers. Any product that may be evaluated in this article, or claim that may be made by its manufacturer, is not guaranteed or endorsed by the publisher.

## Supplementary material

Supplementary Figure 1 The MRI images were taken at baseline of cancer diagnosis, after surgery and at recurrence. **(A)** In April 2014, brain lesions were found following an MRI scan of the patient. **(B)** After surgery, no lesions were found on follow-up in April 2015. **(C, D)** The right temporal **(C)** and occipital **(D)** recurrence in November 2015. The white arrow represents the lesion.

Supplementary Figure 2 DNA NGS results for lung lesion. **(A)** Integrative Genomic Viewer (IGV) snapshot showing KRAS mutation (V14I). **(B, C)** IGV snapshot showing TP53 mutations (R213* and R273H). **(D)** IGV snapshot showing FGFR3-TACC3 fusion.
