# Supplementary figures and images for: Case report: The effective response to pembrolizumab in combination with bevacizumab in the treatment of a recurrent glioblastoma with multiple extracranial metastases
Source: Front Oncol. 2022 Aug 16;12:948933. doi: 10.3389/fonc.2022.948933 (PMC9424992; doi:10.3389/fonc.2022.948933)

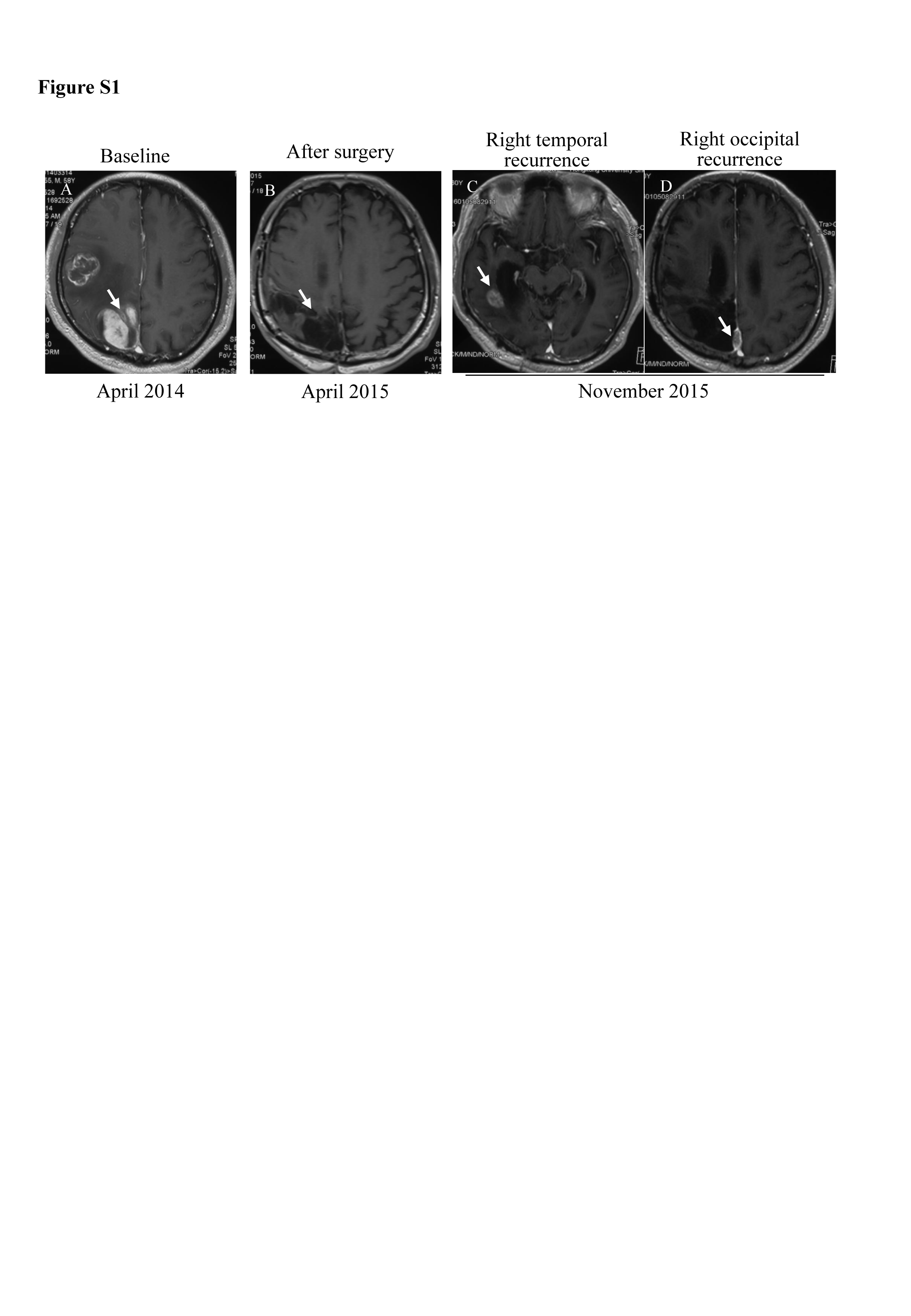

Supplement: Supplementary Figure 1 — The MRI images were taken at baseline of cancer diagnosis, after surgery and at recurrence. (A) In April 2014, brain lesions were found following an MRI scan of the patient. (B) After surgery, no lesions were found on follow-up in April 2015. (C, D) The right temporal (C) and occipital (D) recurrence in November 2015. The white arrow represents the lesion. [file Image_1.tif]

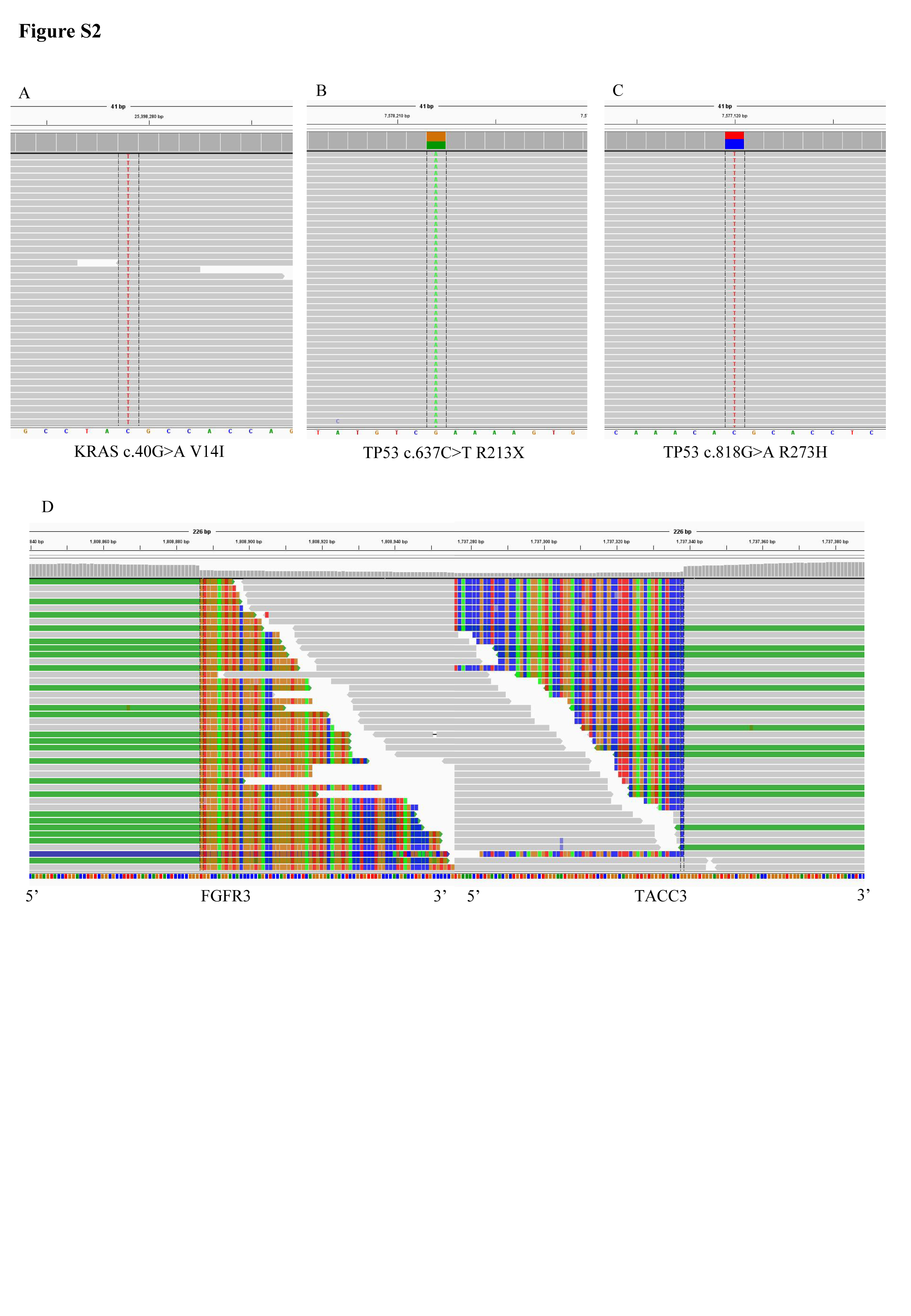

Supplement: Supplementary Figure 2 — DNA NGS results for lung lesion. (A) Integrative Genomic Viewer (IGV) snapshot showing KRAS mutation (V14I). (B, C) IGV snapshot showing TP53 mutations (R213* and R273H). (D) IGV snapshot showing FGFR3-TACC3 fusion. [file Image_2.tif]
